# Pyruvate accumulation may contribute to acceleration-induced impairment of physical and cognitive abilities: an experimental study

**DOI:** 10.1042/BSR20204284

**Published:** 2021-04-14

**Authors:** Fengfeng Mo, Hongwei Zhang, Yuxiao Tang, Ruirui Qi, Shuang Nie, Hui Shen, Min Li

**Affiliations:** 1 Department of Nutrition and Food Hygiene, Faculty of Navy Medicine, Naval Medical University, 800 Xiangyin Road, Shanghai 200433, China; 2 Department of Nutrition, Second People’s Hospital of Zhumadian City, Henan Province 100191, China

**Keywords:** acceleration exposure, insulin, physical and cognitive abilities, pyruvate

## Abstract

**Background:** Fatigue can be induced after acceleration exposure, however its mechanism is still unclear. The aim of the present study was to examine whether metabolites’ changes can decrease cognitive and physical function after acceleration. **Methods:** Graybiel scale and Fatigue Self-rating scale were used to assess the seasickness and fatigue degrees of 87 male seafarers respectively after sailing. To test the effect of pyruvate on cognitive and physical functions, five different doses of pyruvate were administrated into rats. Insulin can reduce the accumulation of pyruvate. To observe the insulin effect on pyruvate, cognitive and physical functions after acceleration, insulin administration or treatment of promoting insulin secretion was used. Physical and cognitive functions were assessed using open field test (OFT), morris water maze (MWM) and loaded swimming test (LST) in animals. **Results:** Physical and cognitive abilities were decreased obviously, and serum pyruvate increased mostly in human and rats after acceleration. Compared with vehicle group, physical and cognitive abilities were significantly decreased after pyruvate administration. Besides, we found a significant decline in adenosine triphosphate (ATP) concentration and pyruvate dehydrogenase (PDH) activity in the hippocampus, prefrontal cortex, liver, and muscle of rats treated with acceleration or pyruvate injection, while insulin administration or treatment of promoting insulin secretion markedly alleviated this decline and the impairment of physical and cognitive abilities, compared with the control group. **Conclusion:** Our results indicate that pyruvate has a negative effect on physical and cognitive abilities after acceleration. Insulin can inhibit pyruvate accumulation and cognitive and physical function after acceleration exposure.

## Introduction

Motion sickness (MS) could be induced by abnormal acceleration. The primary signs and symptoms of MS include nausea, vomiting, drowsiness, and sweating [1]. Many studies have shown that human performance and cognitive tasks are substantially impaired during severe MS [2]. So far, pharmacologic and behavioral strategies are often used to prevent MS. Scopolamine (Sco) is currently the main anti-MS drug, but it may induce remarkable and continuous decrements in work efficiency and extreme fatigue [3]. Habituation strategy is also widely used for relieving MS symptoms, however, there are evidences showing that it can not effectively prevent acceleration-induced fatigue [4].

It has been reported that homeostasis can be disturbed by unfamiliar acceleration [5]. Myburgh et al. found various metabolites might accumulate during fatigue [6]. A prior review stated that several amino acids absence may act as a key factor for exercise performance impairment induced by metabolic acidosis [7]. Unfortunately, relatively little attention has been paid to the etiology and pathogenesis of cognitive and physical performance decline caused by abnormal acceleration exposure. The precise pathophysiologic mechanisms underlying this are still unclear.

Metabonomics is a powerful tool to quantitatively identify dynamic multi-parametric metabolic changes of organism after pathophysiological stimuli [8]. Ultra-high performance liquid chromatography coupled to quadrupole time-of-flight mass spectrometry (UPLC-QTOF-MS/MS) techniques are considered to be suitable for metabonomics due to its high sensitivity in detecting metabolites. Metabolites in blood can reflect the normal physiological changes of the body and the pathophysiological effects of diseases. Analysis of metabolic changes in different individuals can help elucidate the underlying mechanisms of diseases. UPLC-QTOF-MS/MS-based metabonomics could also be used to explore the mechanisms of acceleration-induced fatigue.

Therefore, we analyzed whether there is a connection between fatigue and MS through human experiments and compared the changes of serum metabolites in rats after acceleration exposure with a UPLC-QTOF-MS/MS-based metabonomics approach and carried out another animal experiment to explore the pathophysiologic mechanisms of decline in cognitive and physical abilities induced by acceleration exposure.

## Materials and methods

### Human protocol

#### Subjects

To evaluate the relationship of MS and fatigue, questionnaires were collected at a military academy after sailing for 3 h in the Sea State Six, including 87 male seafarers (age: 24.87 ± 2.36 years, BMI: 22.02 ± 1.57, body weight: 64.61 ± 5.41 kg). Graybiel scale [9] was used to determine severity of MS in cadets at the end of voyage and Fatigue Self-rating Scale [10] was used to assess the fatigue degree at the end of the same voyage.

Informed consents were obtained from all volunteers in the present study and the study protocol was approved by Committee on Ethics of Biomedicine, Naval Medical University (Reference no: 2009LL010). Following an explanation of the entire procedure, all participants provided written informed consent to participate in the study.

### Animal protocol

#### Animals

A total of 384 male Sprague–Dawley rats, weighing 250–300 g, were purchased from Sino-British SIPPR/BK Lab Animal Ltd (Shanghai, China). All animal experiments took place at Naval Medical University (Shanghai, China). Rats were held in cages (four rats per cage) at temperature: 24 ± 2°C and a 12 h light–12 h dark cycle (lighting: 8:00–20:00). Food and water were available *ad libitum*, except during experiments. All animals were acclimated to the lab environment for 1 week. The animals were anesthetized with intraperitoneal injection of 2% pentobarbital sodium 40 mg/kg, which offered good analgesic potency and sedation for rats. Our experiments were conducted according to the National Institutes of Health ‘Guide for the Care and Use of Laboratory Animals’ as well as the guidelines of the Animal Welfare Act. All efforts were made to minimize the number and suffering of rats. The protocol was approved by Committee on Ethics of Biomedicine, Naval Medical University (Shanghai, China) (Reference no: 2009LL010).

### Experiment I: The changes in metabolites and cognitive and physical functions after acceleration

Sixty-four rats were randomly divided into two groups (*n*=32 per group). (1) **Control**: rats were transferred into the containers of the acceleration device without rotation for 2 h. (2) **Exposure**: rats received 2-h acceleration.

### Experiment II: The minimum dose of pyruvate administration to affect cognitive and physical functions

A total of 160 rats were randomly divided into five groups (*n*=32 per group) to confirm the minimal dose to induce fatigue. (1) **Vehicle**: rats were administrated 0.9% sterile saline solution intraperitoneally (i.p.; 1.5 ml per rat). (2) **250 mg/kg**: rats were treated with 250 mg/kg pyruvate dissolved in 0.9% sterile saline solution i.p. (3) **500 mg/kg**: rats were treated with 500 mg/kg pyruvate i.p. (4) **700 mg/kg**: rats were treated with 700 mg/kg pyruvate i.p. (5) **1000 mg/kg**: rats were treated with 1000 mg/kg pyruvate by i.p. Stress-related hormones levels, Adenosine triphosphate (ATP) and Pyruvate dehydrogenase (PDH) activity, in the groups which was injected by the minimum dose of pyruvate to affect cognitive and physical functions were measured.

### Experiment III: The insulin effect on pyruvate administration and cognitive and physical functions after acceleration

A total of 160 rats were randomly divided into five groups (*n*=32 per group). (1) **Control**: rats were administrated with 0.9% sterile saline solution (1.5 ml/rat), by intragastric administration 30 min before transferring into the containers of the acceleration device without rotation for 2 h. (2) **Exposure**: rats were administrated with 0.9% sterile saline solution (1.5 ml/rat), by intragastric administration 30 min before 2-h acceleration. (3) **Exposure+Sco**: rats were administrated with Scopolamine (1 mg/kg), dissolved in 0.9% sterile saline solution (1.5 ml/rat), by intragastric administration 30 min before 2-h acceleration. (4) **Exposure+Ins**: rats received intraperitoneal injection of insulin 30 min before 2-h acceleration (1 unit/kg, Actrapid human insulin, Novo Nordisk, Bagsvaerd, Denmark). (5) **Exposure+protein combined with carbohydrate (CPL)**: rats were administrated with glucose (4.8 g/kg), maltose (2.4 g/kg), casein (1.8 g/kg) and leucine (0.6 g/kg), dissolved in 0.9% sterile saline solution (1.5 ml/rat), by intragastric administration 30 min before 2-h acceleration.

### Acceleration treatment

The exposure device and detailed methods have been described by Cai et al. previously [11]. Briefly, animals were placed in plexiglass containers with the long axis of the body perpendicular to the horizontal rotation rod. The device started to rotate in a clockwise direction at 16°/s^2^ to reach an angular velocity of 120°/s^2^ and then began to decelerate at 48°/s^2^ to reach 0°/s^2^. After a 1-s pause, the container continued to rotate in a counterclockwise direction in the same manner as above. The clockwise-pause-counterclockwise cycle lasted for approximately 21 s.

After acceleration, eight rats in each group underwent open field test (OFT) test, eight rats in each group underwent morris water maze (MWM) test, another eight rats in each group underwent loaded swimming test (LST) and the remaining rats in each group were killed by anesthesia with ketamine (100 mg/kg, i.p.), and the blood was immediately collected using BD Vacutainer tubes (Becton Dickinson Medical Devices, Shanghai, China). Blood samples were allowed to clot for 1 h at 4°C and then centrifuged for 20 min at 1400×**g**. The supernatant was collected. After perfusion with normal saline, the liver, hindlimb muscles, hippocampus, and prefrontal cortex were collected, wrapped, and immediately immersed in liquid nitrogen. All samples were then immediately frozen and kept at −80°Cuntil further analysis.

### Chemicals and reagents

Methanol and acetonitrile (ACN) were obtained from Merck (Darmstadt, Germany). Formic acid was purchased from Fluka (Buchs, Switzerland). Lysine, Methionine, Tryptophan, Histidine, Isoleucine, Creatinine, Phenylalanine, Threonine, Valine, Proline, Serine, Arachidonic acid, Glucose, β-Hydroxybutyric acid, Pyruvic acid (Pyruvate) were obtained from Shanghai Jingchun Chemical Reagent Co.

### Serum sample preparation and serum metabolites acquisition

A 100-μl aliquot of serum sample in control and exposure groups was obtained, then 300 μl of methanol was added to precipitate the proteins. The mixed solution was shook for 1 min and spun at 13000 rpm for 15 min at 4°C. The clear supernatant (100 μl) was transferred to a sampling vial for UPLC-QTOF-MS/MS analysis. Meanwhile, 10 μl aliquot of each sample was also transferred to a sampling vial and mixed as a quality control (QC) to check the stability of the system and method.

An Agilent 6520 UPLC-QTOF MS/MS (Agilent, U.S.A.) was used in the study. Chromatographic separations were performed on an XSELECT™ HSS T3 column (2.1 mm × 100 mm × 2.5 mm, Waters) at a column oven temperature of 40°C; 0.1% formic acid (Reagent A) and ACN (Reagent B) were used as the mobile phase. The gradient conditions were as follows: 0–2 min, 5% B; 2–17 min, 5–95% B; 17–20 min, 95% B; 20–21 min, 95% B, and the post time was 6 min for column equilibration. The flow rate was maintained at 0.4 ml/min and the injection volume were 4 μl. The auto-sampler temperature was set at 4°C. An electrospray ionization source (ESI) was set in both positive and negative ion modes, and mass spectrometric parameters were performed as follows: the scanning range was set at 50–1100 m/z, electrospray capillary voltage with 4000 V in positive mode and 3.5 kV in negative mode were used, the nebulizer pressure was set at 45 psi, nitrogen was used as drying gas with the flow rate of 11 l.min^−1^ and the temperature of 350°C, the fragment voltage was maintained at 120 V, skimmer voltage was set at 60 V, Octopole RF Peake was set at 750 V, 121.0509 and 922.0098 Da were used at reference masses (m/z). QC samples were randomly applied to into the sequence of samples. The biomarker candidates were subsequently identified by MS/MS, and the collision energy was set 10, 20, and 40eV.

### Data reduction and pattern recognition

All data were acquired using Agilent Mass Hunter workstation software version B.01.04 (Agilent, MA, U.S.A.). Firstly, the UPLC-QTOF MS/MS raw data (.d) were converted into mzdata files. The isotope interferences were excluded and the threshold of the absolute peak height was set at 500. The R package ‘xcms’ was employed to generate a data matrix through peak extraction, alignment and integration and forma visual table including sample names, and peak indexes (m/z-Rt pairs and peak area). XCMS parameters were default settings except for the following: fwhm = 8, bw = 10, and snthersh = 5. All the ions were filtered based on the 80% rule before all of the detected ions in each sample were normalized to the sum of the peak area to obtain the relative intensity of metabolites based on MATLAB7.1 (The MathWorks, Inc., U.S.A.). After being normalized, ion intensities were converted into CSV data and imported into the SIMCA-P program (version 12.0, Umetrics, Umea, Sweden) for principal component analysis (PCA) and Orthogonal partial least-squares-discriminant analysis (OPLS-DA) after mean-centering and Pareto scaling. The parameters (R2X, R2Y, and Q2Y) showing the goodness of fit and prediction were assessed by SIMCA-P for internal validation. The variable importance (VIP) value, generated in OPLS-DA processing, determine the most significant metabolites in rats after acceleration using the VIP value (VIP > 1). The parameters (R2X, R2Y, and Q2Y) were used to determine the goodness of fit and prediction for internal validation.

In our study, a two-tailed Student’s *t* test (GraphPad Prism Inc., La Jolla, CA, U.S.A.) were used to determine the statistical difference between the control and exposure groups.

In order to verify the different metabolites, first ions based on the extracted ion chromatogram were confirmed and then the extracted molecular weight with the common metabolite databases such as the Human Metabolome Database (https://hmdb.ca) and Metlin (http://metlin.scripps.edu) was compared. At the same time, fragment ions were analyzed through MS/MS to narrow the scope of target compounds. Finally, commercial standards were used to support the metabolite identification.

### OFT

The OFT is widely used to assess exploratory behavior in rodents [12]. During OFT, an animal behavior test system (RD1112-IFO-R-4, Mobiledatum, Shanghai, China) was used with a dark cuboid chamber (length: 40 cm, width: 40 cm, height: 90 cm). The apparatus was illuminated by a 100 W bulb that focused on the field with a height of ∼80 cm from the ground. Each rat was placed gently into the center of the open field and allowed to explore the area for 5 min. The chambers ware cleaned between experiments for each rat. The total distance traveled (body center-point) were measured with commercially available software (EthoVision XT 8.5, Noldus, Netherlands).

### MWM

MWM was used to assess the ability of cognitive function in rats [13]. MWM was a cylindrical, black-painted pool (1.5 m diameter, 0.6 m height) filled with water (0.4 m depth, temperature: 23–24°C) and divided into Northeast, Southeast, Southwest, and Northwest quadrants. Four different posters of shapes and colors were placed around the black wall enclosure to define the site of the platform. A black-painted platform (10 cm diameter, 1 cm below the water surface) was placed in the determinate quadrant. The animals were permitted to swim for 90 s until they found the platform and remained on it for 10 s. If unsuccessful in 90 s, the rat was gently guided to the platform by the experimenter and allowed to remain on it for 30 s. The position of the platform remained unchanged. Four trials were conducted for each rat for the spatial learning task with an intertribal interval of 5 min. The rats received three consecutive daily trainings. The time to find the platform were expressed as an escape latency and recorded using commercially available software (EthoVision XT 8.5, Noldus, Netherlands).

### LST

The loaded swimming capacity test was employed in our study to evaluate physical durability of rats [14]. The physical capacity was measured with acrylic plastic pool (100 cm × 45 cm × 45 cm) filled with water to a depth of 85 cm. The temperature of the water was maintained at 23–24°C. The rats were loaded with a steel washer weighing 4% of their body weight attached to the tail. The swimming time (min) to exhaustion was used as the index of the forced swimming capacity. The rats were assessed to be exhausted when they failed to rise to the surface of water to breathe within a 10-s period.

The time of the animals subjected to OPT, MWM, and LST was 18:00–22:00.

### Serum hormone determination

Serum insulin, glucagon, and cortisol levels were measured by radioimmunoassay kits of each hormone (North Institute of Biological Technology Co, Beijing, China). The concentration was calculated by regression analysis of a standard curve according to the instructions of the manufacturers. Adrenaline and Norepinephrine was analyzed by immunoenzyme assay (commercial kits, Nanjing Jiancheng Biotechnology, China).

### PDH activity assay

PDH activity was measured in the tissues according to manufacturer’s instructions (Abcam, Inc, Cambridge, MA, U.S.A.). Intact PDH was solubilized by adding 1 volume of detergent to 19 volume of each sample, incubated on ice for 10 min, then 12000×***g*** centrifuged for 10 min at 4°C. The supernatant was collected and the PDH enzyme was immunocaptured within each well by PDH antibodies and subjected to functional activity and quantitative microplate assays (Abcam, Inc, Cambridge, MA, U.S.A.). Absorbance of each well was measured at 450 nm on a microplate reader (synergy2, BioTekInc, VT, U.S.A.) at room temperature using a kinetic program for 20 min. PDH activity was expressed as the initial rate of reaction, determined from the slopes of the curves generated.

### Detection of ATP levels

Tissue ATP levels were measured using a firefly luciferase-based ATP assay kit (Beyotime Institute of Biotechnology, Shanghai, China) according to the manufacturer’s instructions. After the indicated treatments, tissues were lysed and centrifuged at 12000×***g*** for 5 min. A 20-μl aliquot of supernatant was mixed with 100 μl of ATP detection working dilution in a black 96-well plate (Costar 3603 Corning, New York, U.S.A.). Luminance (RLU) was measured by using a microplate reader (synergy2, BioTekInc, VT, U.S.A.). Standard curves were also generated and the protein concentration of each sample was determined using the Pierce™ BCA Protein Assay Kit (Thermo Fisher Scientific Inc, Massachusetts, U.S.A.). Total ATP levels were expressed as nmol/mg protein.

### Statistical analysis

The GraphPad Prism v6.0 (GraphPad Prism Inc., La Jolla, CA, U.S.A.) was used to analyze and present all the data. Multiple comparisons were performed using one-way ANOVA, followed by Tukey’s post-hoc test. The correlation coefficient between the scores of fatigue and MS in human were carried out using Pearson correlation coefficient. Statistical significance level was set at 0.05.

## Results

### Fatigue and seasickness can be induced after sailing and the degree of seasickness is markedly correlated with the fatigue degree

**Figure 1:**
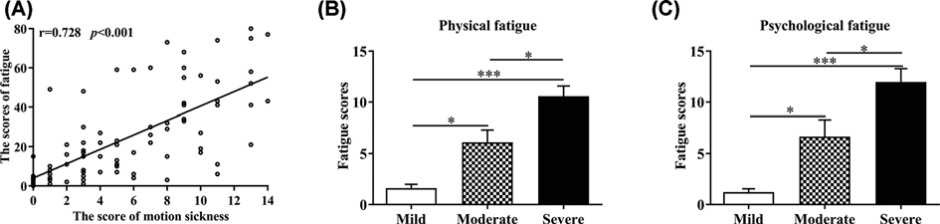
The degree of fatigue and seasickness in 87 male seafarers after sailing (**A**) The score of MS in 87 male seafarers after sailing. (**B**) The degree of physical fatigue in 87 male seafarers after sailing. (**C**) The degree of psychological fatigue in 87 male seafarers after sailing. Data from each group were expressed as mean ± SEM in histogram. Significantly different at *P<0.05, ***P<0.001 vs each group.

We investigated the degree of fatigue and seasickness in 87 male seafarers after sailing 3 h in Sea State Six. A total of 100% seafarers got fatigue and 90.8% were at varying degrees of seasickness, among which 15, 18, and 46 participants got mild, moderate, and severe seasickness, respectively. As shown in Figure 1A, there was a significant positive correlation (r = 0.728, *P*<0.001) between the degrees of seasickness and fatigue. Further analysis found that both mental fatigue (*F* (2, 84) = 20.99, *P*<0.05, Figure 1B) and physical fatigue scores (*F* (2, 84) = 17.45, *P*<0.001, Figure 1C) were increased, with the increase in the seasickness degree in seafarers. Results indicated that mental and physical fatigue of seafarers is related to acceleration exposure after sailing.

### Metabolite changes after acceleration exposure in rats

**Figure 2:**
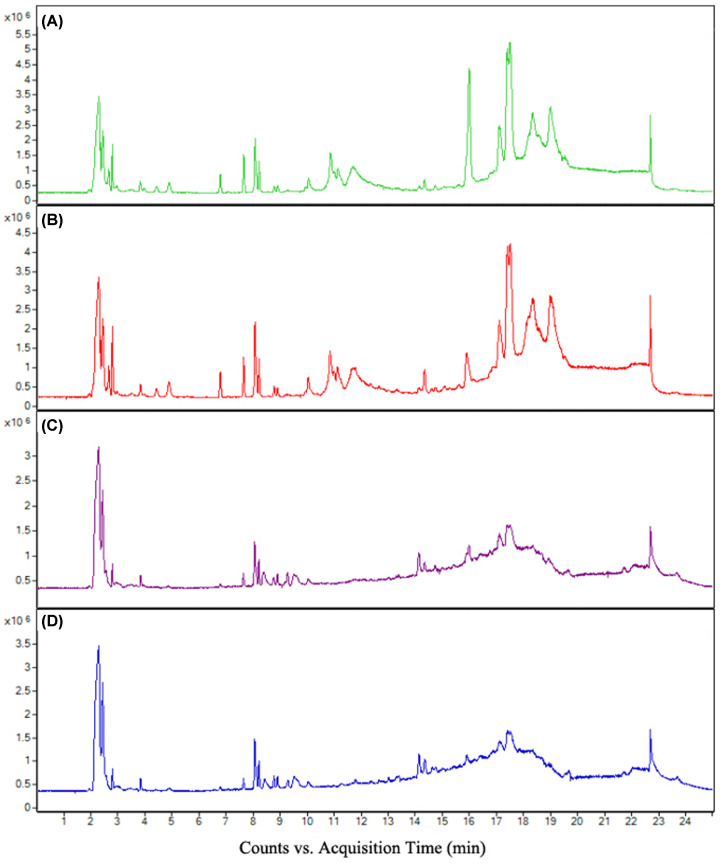
The UPLC-QTOF-MS/MS total ion current chromatograms of serum samples in different modes between control and exposure groups (**A**) The UPLC-QTOF-MS/MS total ion current chromatograms of serum samples in positive mode in control group. (**B**) The UPLC-QTOF-MS/MS total ion current chromatograms of serum samples in positive mode in exposure group. (**C**) The UPLC-QTOF-MS/MS total ion current chromatograms of serum samples in negative mode in control group. (**D**) The UPLC-QTOF-MS/MS total ion current chromatograms of serum samples in negative mode in exposure group.

**Figure 3:**
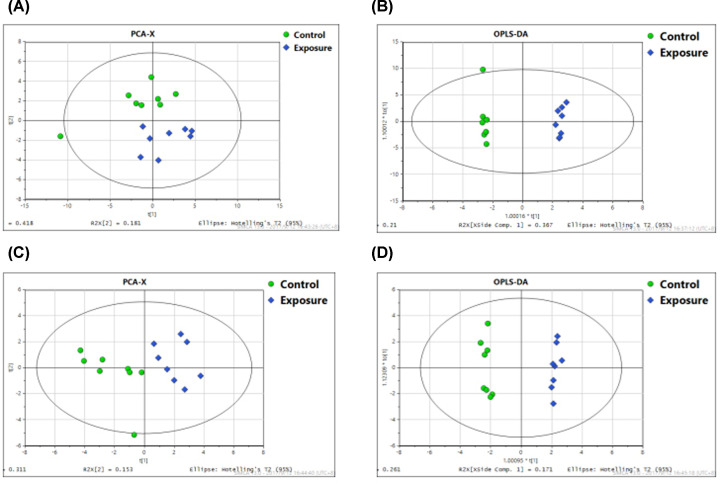
Plots of PCA and OPLS-DA of serum samples applied from the control group and acceleration exposure group in ESI positive and negative ion modes (**A**) Plots of PCA of serum samples applied from the control group in ESI positive ion mode. (**B**) Plots of OPLS-DA of serum samples applied from the control group in ESI positive ion mode. (**C**) Plots of PCA of serum samples applied from the control group in ESI negative ion mode. (**D**) Plots of OPLS-DA of serum samples applied from the control group in ESI negative ion mode.

In our previous studies, 60 male volunteers were recruited to analyze the causes of physical and mental fatigue induced by acceleration [15]. We found acceleration-induced significant changes of serum metabolites. Among the 78 metabolites identified by OPLS-DA model, 35 metabolites were significantly changed after acceleration exposure. Six metabolites were changed over twice, among which the pyruvate level increased most rapidly (4.09-fold). Animal experiments were employed to verify the results of the human experiments. Figure 2 shows the UPLC-QTOF-MS/MS total ion current chromatograms of serum samples in the ESI positive and negative ion modes between control and exposure groups: (A,C) control group, (B,D) exposure group in rats. The shape of each peak is good and the peaks are separated well, indicating that the chromatographic and mass spectrometry conditions were appropriate to measure samples in the present study. A summary of all the observations were based on PCA. An obvious difference between the control and exposure groups was observed in the PCA score plot of positive and negative models (Figure 3A,C). OPLS-DA was subsequently performed to identify the most significant metabolites that can discriminate the metabolic distinction (Figure 3B,D). The R2X, R2Y, and Q2Y value in positive ion model was 0.716, 0.996, and 0.924, while The R2X, R2Y, and Q2Y value in negative ion mode was 0.533, 0.991, and 0.903, indicating that the model had a good predictive power and goodness of fit. Combining the VIP values from the OPLS-DA model with the results from the two-tailed Student’s *t* test (*P*<0.05), 25 metabolites were selected as differential metabolites in positive and negative model, respectively (Table 1).

**Table 1:** Changes in serum part metabolites in rats after acceleration exposure

Metabolite	FC	*P*-value	Change	Metabolite	FC	*P*-value	Change
4-Hydroxy-l-proline^3^	0.77	0.0167	Down	Serine^1^	0.83	0.0021	Down
Lysine^1^	0.50	0.0009	Down	Arachidonic acid^1^	1.20	0.0043	Up
Methionine^1^	0.37	0.0013	Down	Glucose^1^	1.12	0.0000	Up
Tryptophan^1^	0.45	0.0000	Down	n-Tetradecanoic acid^3^	1.32	0.0072	Up
Histidine^1^	0.38	0.0306	Down	Stearic acid^2^	1.43	0.0119	Up
Isoleucine^1^	0.77	0.0107	Down	Palmitic acid^2^	1.60	0.0004	Up
Creatinine^1^	0.91	0.0108	Down	Elaidic acid^2^	1.30	0.0096	Up
Phenylalanine^1^	0.53	0.0009	Down	n-Dodecanoic acid^3^	1.60	0.0004	Up
Threonine^1^	0.71	0.0103	Down	Linoleic acid^2^	1.21	0.0116	Up
Carbodiimide^3^	0.42	0.0000	Down	Oleic acid^2^	2.70	0.0058	Up
Valine^1^	0.67	0.0005	Down	β-Hydroxybutyric acid^1^	1.21	0.0013	Up
Proline^1^	0.67	0.0000	Down	Pyruvic acid (Pyruvate)^1^	3.20	0.0000	Up
Urea^2^	0.67	0.0000	Down				

Fold changes (FC) were obtained by comparing the mean concentration of each metabolite in exposure group with that in control group; FC value greater than 1 indicate a higher concentration (Up) after acceleration exposure. The value less than 1 indicate a lower concentration (Down) after acceleration exposure (*n*=8 per group).

1 The metabolites were verified by standard compound.

2 Metabolites analyzed based on MS/MS chromatograms.

3 Metabolites putatively annotated.

### The cognitive and physical abilities of rats significantly decreased after acceleration exposure, meanwhile serum pyruvate level elevated and insulin level decreased

In order to see whether the accumulation of pyruvate *in vivo* after exposure is related to the occurrence of physical and mental fatigue, OFT, MWM and LST were performed to examine the changes of cognitive and physical function of rats after acceleration.

**Figure 4:**
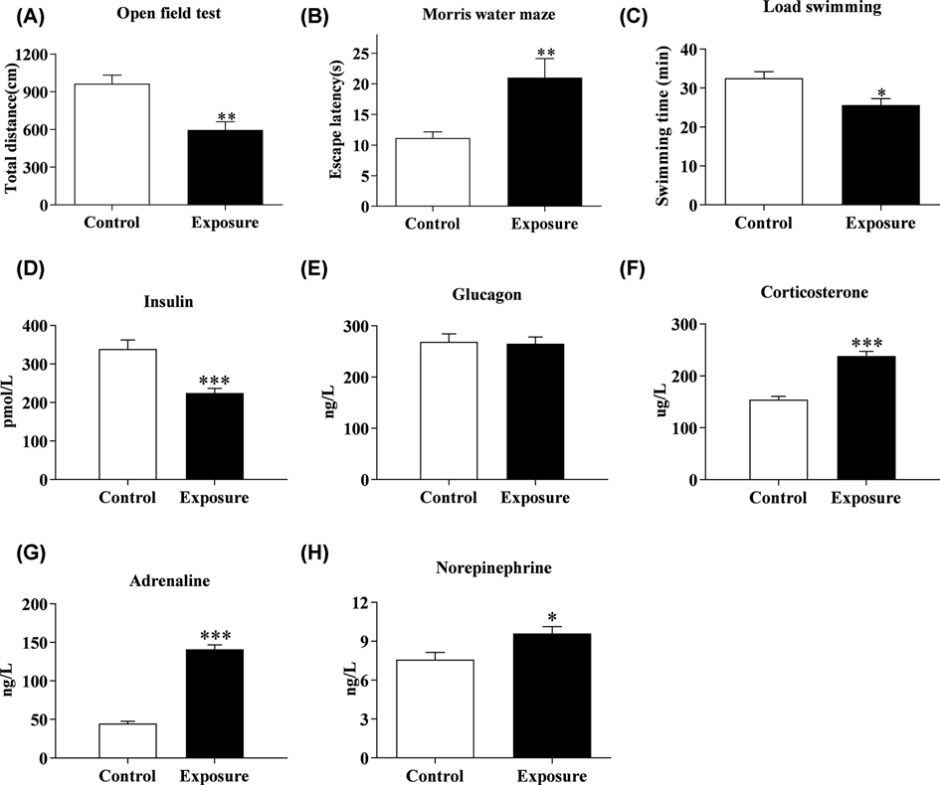
The changes of cognitive and physical capacity and hormones levels in rats after acceleration exposure (**A**) Changes of total distance traveled in rats after acceleration exposure. (**B**) Changes of escape latency in rats after acceleration exposure. (**C**) Changes of swimming time in rats after acceleration exposure. (**D**) Changes of insulin in rats after acceleration exposure. (**E**) Changes of the glucagon in rats after acceleration exposure. (**F**) Changes of corticosterone in rats after acceleration exposure. (**G**) Changes of adrenaline in rats after acceleration exposure. (**H**) Changes of norepinephrine in rats after acceleration exposure. Data from each group were shown as mean ± SEM in histogram. Significantly different at *P<0.05, **P<0.01, ***P<0.001 vs control. N=10 per group.

A significant decline of physical and cognitive function was observed in exposure group. The escape latency increased by 86% (*P*<0.01) in the exposure group, while total distance traveled and swimming time decreased by 35% (*P*<0.01) and 24% (*P*<0.05) obviously in the exposure group compared with those of control group (Figure 4A–C). Results also showed that pyruvate level was 3.2-fold higher in exposure group compared with the control group (Table 1). The concentration of insulin decreased, while corticosterone, adrenaline and norepinephrine levels increased markedly in the exposure group, compared with the control group (*P*<0.001 for insulin; *P*<0.001 for corticosterone, *P*<0.001 for adrenaline, *P*<0.05 for norepinephrine) (Figure 4D–H).

Results showed that rats’ cognitive and physical abilities were markedly impaired by acceleration exposure which can also induce the increase in serum pyruvate level and the decrease in insulin level significantly at the same time.

### Accumulation of pyruvate is a key factor for the decrease in cognitive and physical function in rats

Compared with the vehicle group, indicators of OFT and LST were significantly shortened, while the indicator of MWM was enhanced after the injection of pyruvate (500, 700, and 1000 mg/kg). Compared with vehicle group, injection of pyruvate at a dose of 250 mg/kg did not affect the movement distance, escape latency, and weight-bearing swimming time of rats. Therefore, we chose a concentration of 500 mg/kg in the follow-up experiment (Supplementary Figure S1) (*F* (4, 19) = 11.79, *P*<0.0001, Supplementary Figure S1A. *F* (4, 55) = 10.73, *P*<0.0001, Supplementary Figure S1B. *F* (4, 19) = 23.67, *P*<0.0001, Supplementary Figure S1C).

**Figure 5:**
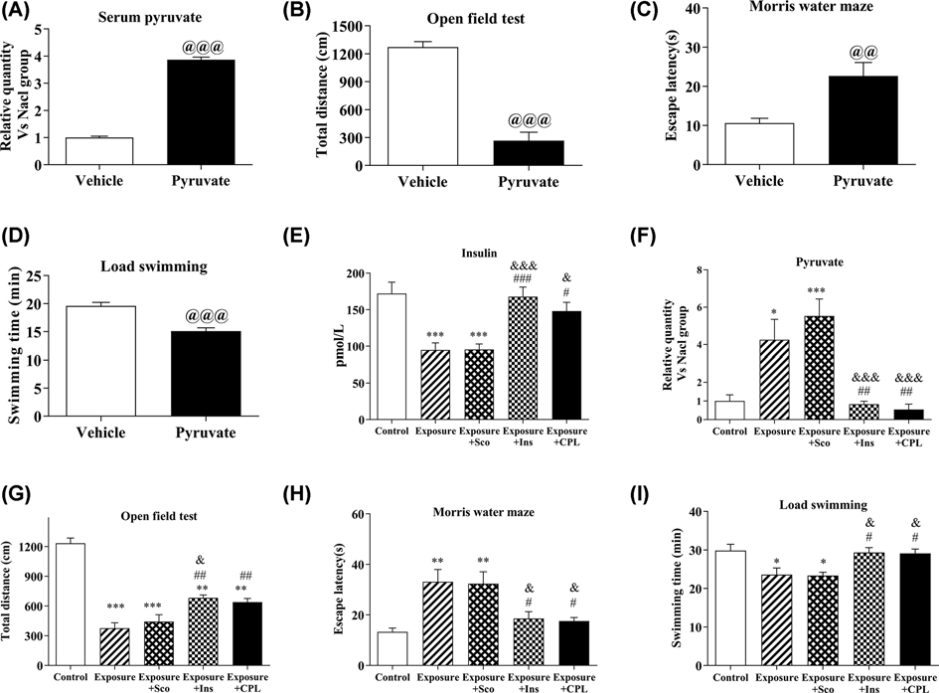
Changes of serum pyruvate level and cognitive and physical capacity in rats after pyruvate injection and different treatment (**A**) Changes of serum pyruvate level in rats after pyruvate injection. (**B**) Changes of total distance traveled in rats after pyruvate injection. (**C**) Changes of escape latency in rats after pyruvate injection. (**D**) Changes of swimming time in rats after pyruvate injection. (**E**) Changes of insulin concentration in rats after different treatments. (**F**) Changes of serum pyruvate level in rats after different treatments. (**G**) Changes of total distance traveled in rats after different treatments. (**H**) Changes of escape latency in rats after different treatments. (**I**) Changes of swimming time in rats after different treatments. Data from each group were expressed as mean ± SEM. Significantly different： *P<0.05, **P<0.01, ***P<0.001 vs control group. #*P*<0.05, ##*P*<0.01, ###*P*<0.001 vs Exposure group. @@*P*<0.01, @@@*P*<0.001 vs vehicle. &*P*<0.05, &&&*P*<0.001 vs Exposure + Sco group in E, F, G, H and I. N=8 per group.

There was a significant increase in serum pyruvate level after 500 mg/kg of pyruvate i.p. (Figure 5A). The cognitive and physical function of rat significantly declined after 500 mg/kg pyruvate injection. There was a significant effect of pyruvate accumulation increasing escape latency (*P*<0.01) in MWM and decreased in both total distance (*P*<0.001) traveled in OFT and swimming time (*P*<0.001) in LST when compared with those in vehicle group (Figure 5B–D).

In the classic theory of fatigue, pyruvate, as a cause of fatigue, can lead to acidosis. A large number of studies have shown that supplementation of insulin can significantly reduce the accumulation of pyruvate in the body [16]. Because of the effect of exogenous insulin on the improvement of brain function after exposure to acceleration [15], a more practical intervention is to increase the amount of insulin before exposure to acceleration. It has recently been reported that protein combined with carbohydrates can effectively improve insulin secretion in the body, which has been confirmed by Nuttal et al. [17]. Van et al. [18] explored the use of multiple amino acids in combination with carbohydrates to promote insulin secretion in clinical and motor environments. In these studies, it was found that protein combined with carbohydrates significantly increased the level of insulin in the body [19]. Therefore, we investigated the effects of exogenous and endogenous insulin on fatigue induced by accelerated exposure by preliminarily administering CPL in rats in the present study.

Our results showed that can serum insulin can be significantly increased (*F* (4, 45) = 10.21, *P*<0.0001, Figure 5E) and the accumulation of serum pyruvate can be reduced by both the Exposure+Ins and Exposure+CPL group after acceleration exposure (*F* (4, 45) = 11.84, *P*<0.0001, Figure 5F). Meanwhile the decline of cognitive and physical function induced by acceleration was also alleviated (*F* (4, 40) = 44.37, *P*<0.0001, Figure 5G. *F* (4, 54) = 7.098, *P*<0.001, Figure 5H; *F* (4, 55) = 5.708, *P*<0.001, Figure 5I). Namely, the escape latency increased while the total distance traveled and swimming time decreased significantly in both Exposure and Exposure+Sco groups when compared with control group. Besides, there was a statistic difference in the total distance, escape latency and swimming time between the control and the Exposure+Ins or Exposure+CPL group. However, there was no significant difference in terms of the total distance, escape latency and swimming time between exposure and exposure+Sco groups.

Results indicated that the pyruvate accumulation may be a key contributory factor for the cognitive and physical function decline in rats induced by acceleration exposure. Insulin can inhibit pyruvate accumulation during acceleration exposure.

### Accumulation of pyruvate *in vivo* can reduce ATP levels in tissue by inhibiting PDH activity

There is controversy about the role of pyruvate supplementation in cognition and physical function. It has been reported that supplemented with calcium pyruvate before exercise can effectively relieve fatigue after exercise [20]. Recently, Koh-Banerjee et al. confirmed that pyruvate supplementation during training did not significantly affect body exercise performance and may negatively affect some blood lipid levels [21]. Pyruvate metabolism is regulated by a variety of key enzymes. PDH plays an important role in the process of pyruvate into tricarboxylic acid cycle. If PDH activity is impaired, pyruvate accumulation is easy to occur and then lead to abnormal energy metabolism. We tested the effect of pyruvate on the levels of ATP concentration and PDH activity in hippocampus, prefrontal cortex, liver and muscle tissues of rats.

**Figure 6:**
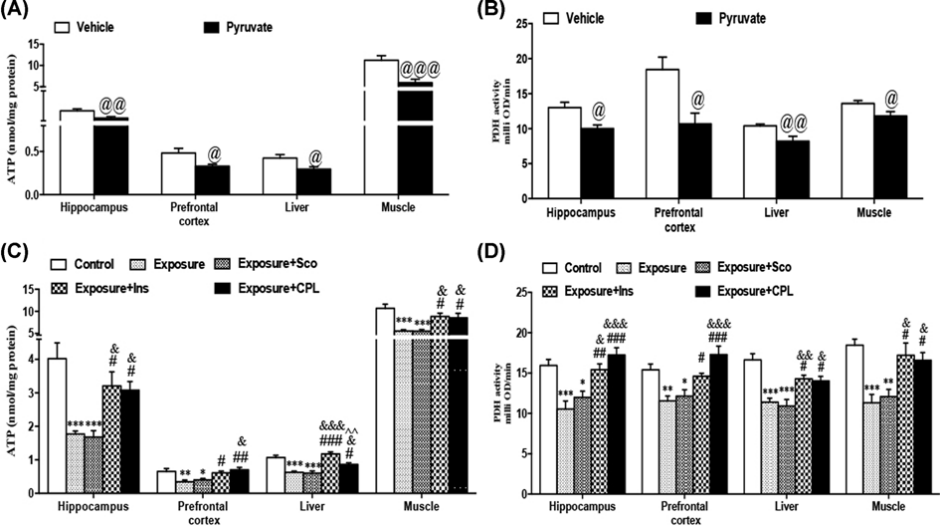
Changes of ATP concentration and PDH activity in hippocampus, prefrontal cortex, liver and muscle of the animals in different groups (**A**) Changes of ATP concentration in hippocampus, prefrontal cortex, liver and muscle of the animals in vehicle and pyruvate-treated groups. (**B**) Changes of PDH activity in hippocampus, prefrontal cortex, liver and muscle of the animals in vehicle and pyruvate-treated groups. (**C**) Changes of ATP concentration in hippocampus, prefrontal cortex, liver and muscle of the animals in control and exposure-treated groups. (**D**) Changes of PDH activity in hippocampus, prefrontal cortex, liver and muscle of the animals in control and exposure-treated groups. Data from each group were expressed as mean ± SEM. Significantly different： *P<0.05, **P<0.01, ***P<0.001 vs control group. #*P*<0.05, ##*P*<0.01, ###*P*<0.001 vs Exposure group. @*P*<0.01, @@*P*<0.01, @@@*P*<0.001 vs vehicle. &*P*<0.05, &&*P*<0.01, &&&*P*<0.001 vs Exposure + Sco group. N=8 per group.

We found that the pyruvate-treated group showed significant reductions in the level of PDH activity and ATP concentration in hippocampus, prefrontal cortex, liver and muscle tissues when compared with those in vehicle group as shown in (Figure 6A,B). Besides, the exposure-treated group had significant reductions in the levels of PDH activity and ATP content in hippocampus (PDH: *F* (4, 35) = 11.61, *P*<0.0001. ATP: *F* (4, 45) = 9.981, *P*<0.0001), prefrontal cortex (PDH: *F* (4, 34) = 10.15, *P*<0.0001. ATP: *F* (4, 44) = 6.637, *P*<0.001), liver (PDH: *F* (4, 35) = 13.46, *P*<0.0001. ATP: *F* (4, 45) = 19.67, *P*<0.0001) and muscle (PDH: *F* (4, 35) = 9.045, *P*<0.0001. ATP: *F* (4, 44) = 9.807, *P*<0.0001) of animals in the exposure group rats when compared with those in control group. PDH activity and ATP content in the tissues mentioned above are obviously increase in both Exposure+Ins or Exposure+CPL group (Figure 6C,D).

## Discussion

MS is referred to as the symptoms when people are exposed to abnormal acceleration, including dizziness, headache, cold sweats, nausea, vomiting and other symptoms. The symptoms such as nausea and vomiting can disappear in a short time after out of abnormal acceleration for 50–80% people, but serious fatigue, drowsiness, loss of appetite, and other symptoms can last for several hours or even longer and have a positive correlation with the degree of MS. The persistence of such symptoms can seriously impair the operational capacity of people who got MS, which are unpleasant to passengers during the trip and even dangerous to air, naval, and space staff. Our results showed that there was a significantly positive correlation between the degrees of seasickness and fatigue after abnormal acceleration exposure, which suggest that unfamiliar acceleration exposure can induce physical and mental fatigue in human although that vary in degree. Interestingly, we found that serum pyruvate concentration of 60 volunteers markedly increased (four times) after acceleration exposure during human experiments and pyruvate level was 3.2-fold higher in exposure group compared with the control group during animal experiments.

Some studies have indicated that pyruvate was considered as an acid-induced fatigue substance, and its excessive accumulation in the body can lead to fatigue [22]. Some researchers have found that the appropriate increase in the pyruvate *in vivo* can improve the extreme athletes’ performance [21]. Lee et al. found a single injection of sodium pyruvate (500 or 1000 mg/kg) after onset of reperfusion significantly, decreased mortality, and neuronal cell death in a rat model of transient global ischemia [23]. Elevated insulin levels can effectively reduce the accumulation of pyruvate [19]. Therefore, we improved the insulin level to reduce the accumulation of pyruvate induced by acceleration exposure through directly i.p. insulin or gavage special nutrients, CPL, that can effectively stimulate insulin secretion *in vivo*. Our results showed that either Exposure+Ins or Exposure+CPL group can reduce serum pyruvate accumulation and at the same time increased total distance traveled in OPT and swimming time in LST, decreased escape latency in MWM when compared with those in exposure group or exposure+Sco group animals. Above results showed that the accumulation of pyruvate in the body (three to four times) may be an important reason for the physical and mental fatigue induced by acceleration exposure, while the anti-MS drug-scopolamine had no effect on the decreased physical and cognitive function of animals exposed to acceleration.

A large number of studies have confirmed that the energy requirements of bodies increased significantly under the stress and fatigue conditions [24]. We found the levels of serum glucocorticoids, epinephrine, and norepinephrine were significantly increased, while the content of insulin was significantly decreased after acceleration exposure. This finding indicated that a significant stress response was caused by acceleration exposure. Apart from that, we found that compared with the control group, the intracellular ATP content and PHD activity of the hippocampus, cerebral cortex, liver, and muscle were significantly decreased in the rats with acceleration exposure which also decreased physical and cognitive function, suggesting that the decline of body physical and cognitive induced by acceleration may be related to the inhibiting of energy metabolism. In order to further understand the effect of pyruvate accumulation on the energy metabolism after acceleration exposure, we examined the ATP content and PDH activity of the tissue mentioned above after intraperitoneal injection of pyruvate and found that the levels of ATP content and PDH activity were significantly decreased when compared with those in vehicle group. We used the methods of insulin injection or CPL stimulate insulin secretion to reduce the accumulation of serum pyruvate. Our results showed that both Exposure+Ins and Exposure+CPL groups effectively alleviate the decline of ATP content and PDH enzyme activity induced by serum pyruvate accumulation, increased total distance traveled in OFT and swimming time in LST, decreased escape latency in MWM when compared with those in exposure-treatment group.

In summary, our study showed that the impairment of cognitive and physical function was associated with pyruvate accumulation, which has an inhibitory effect on PDH activity and ATP synthesis in tissue cells, induced by abnormal acceleration. Further functional analyses suggested that insulin can inhibit pyruvate accumulation and cognitive and physical function after acceleration exposure. Longer term studies must be done to elucidate the precise mechanism of how pyruvate accumulation impact ATP metabolism. It would be also interesting to study the mechanism of pyruvate accumulation *in vivo* after acceleration.

## Supplementary Material

Supplementary Figure S1

## Data Availability

Some or all data, models, or code generated or used during the study are available from the corresponding authors on request.

## Abbreviations

ACN: methanol and acetonitrile
ATP: adenosine triphosphate
CPL: protein combined with carbohydrate
ESI: electrospray ionization source
Ins: insulin
LST: loaded swimming test
MS: motion sickness
MWM: morris water maze
OFT: open field test
OPLS-DA: orthogonal partial least-squares-discriminant analysis
PCA: principal component analysis
PDH: pyruvate dehydrogenase
QC: quality control
Sco: scopolamine
UPLC-QTOF-MS/MS: ultra-high performance liquid chromatography coupled to quadrupole time-of-flight mass spectrometry
VIP: variable importance
