# Supplementary material for: Pyruvate accumulation may contribute to acceleration-induced impairment of physical and cognitive abilities: an experimental study
Source: Biosci Rep. 2021 Apr 14;41(4):BSR20204284. doi: 10.1042/BSR20204284 (PMC8047541; doi:10.1042/BSR20204284)
Supplement: Supplementary Figure S1 [file BSR-2020-4284_supp.pdf]

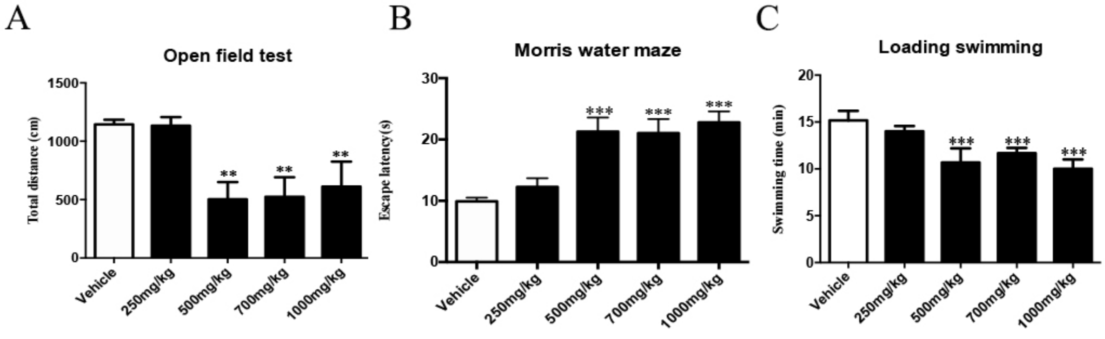


**Supplemental Fig. S1 The minimum dose of pyruvate administration to affect cognitive and physical functions. *p<0.05, **p<0.01, ***p<0.001 vs Vehicle**

**Fig.S1A Changes of total distance traveled in different dose of pyruvate administration**

**Fig. S1B Changes of escape latency in different dose of pyruvate administration**

**Fig. S1C Changes of swimming time in different dose of pyruvate administration**
